# Aripiprazole-Associated Rhabdomyolysis in a 17-Year-Old Male

**DOI:** 10.34172/aim.2023.08

**Published:** 2023-01-01

**Authors:** Ayşe Kutlu, Çisel Yazan Songür, Hurşit Apa

**Affiliations:** ^1^Department of Psychology, Istanbul Esenyurt University, Faculty of Art and Social Sciences, Esenyurt, Istanbul, Turkey; ^2^Department of Child and Adolescent Psychiatry, University of Health Sciences Dr. Behcet Uz Child Disease and Pediatric Surgery Training and Research Hospital, Konak, Izmir, Turkey; ^3^Department of Child Emergency, University of Health Sciences Dr. Behcet Uz Child Disease and Pediatric Surgery Training and Research Hospital, Konak, Izmir, Turkey

**Keywords:** Aripiprazole, Atypical antipsychotics, Case report, Rhabdomyolysis

## Abstract

Rhabdomyolysis is a rare serious side effect of antipsychotic medication use. There are cases of rhabdomyolysis due to the use of clozapine, risperidone, olanzapine, and haloperidol in the literature. In this report, we describe a rhabdomyolysis case developed on the 13th day of using 2.5 mg /day aripiprazole in a 17-year-old male patient with a diagnosis of somatic symptom disorder. This case is one of the youngest in the literature to develop rhabdomyolysis after the use of aripiprazole. Moreover, this case is distinguished from the others with its low-dose, short-term and single antipsychotic use. In the child and adolescent age group, routine blood tests should be done before starting medication. Symptoms that appear to be nonspecific and that may be overlooked or may be thought to be caused by an existing psychiatric complaint should be carefully and thoroughly considered during follow-up.

## Introduction

 In recent years, the use of antipsychotics, especially atypical antipsychotics, has increased in the child and adolescent psychiatry treatment practices.^1^ After the use of antipsychotics; side effects such as weight gain, increased lipid and liver transaminase levels, prolactin levels, and extrapyramidal symptoms may occur frequently.^2^ Rhabdomyolysis is a rare but serious side effect during antipsychotic drug use. Most of the cases reported in the literature are due to chronic drug use. The most common cause of rhabdomyolysis is psychiatric drugs: clozapine, risperidone, olanzapine, and haloperidol.^3^ It is a clinical condition that develops because of the release of cell contents into plasma from muscle breakdown. Drugs, toxins, infections, muscle trauma, seizures, hyperthermia, electrolyte imbalances, muscle enzyme defects, and alcohol use may cause rhabdomyolysis.^4^ The mechanism of rhabdomyolysis due to antipsychotic drug use has not been elucidated yet. However, it is thought to be associated with serotonergic or dopaminergic blockade.^5^

 Rhabdomyolysis due to antipsychotic drugs is more common in adults; there are a few case reports in children and adolescents. Rhabdomyolysis cases that develop with the use of aripiprazole are reported for adult cases and are very rare. The presented patient is found to be one of the youngest cases in the literature to develop rhabdomyolysis as a result of aripiprazole use, at a very low dose and in a short period of time with a single antipsychotic use.^6^

## Case Report

 A 17-year-old male patient consulted with the child and adolescent psychiatry clinic University of Health Sciences Dr. Behcet Uz Child Disease and Pediatric Surgery Training and Research Hospital, Izmir Turkey due to chest pain that started 3 months ago, feeling faint, inability to breathe, and worries about constantly getting sick. The patient, who had thoughts about having a physical illness, came to the emergency service of the hospital whenever he had complaints and had detailed examinations repeatedly before having a psychiatric consult. No pathological findings in medical evaluations and blood tests, and no cardiac and neurological causes were found after further consultations. The patient did not have any previous medical illnesses in his medical history; however, it was learned that the patient’s aunt and cousin had a diagnosis of schizophrenia. Aripiprazole 2.5 mg/d was started for the patient with a diagnosis of somatic symptom disorder. In his control examination one week after the drug was started, it was found that his psychiatric complaints decreased. Our case came to the emergency service with the presentation of complaints such as nausea, dizziness, and weakness on the 13th day of Aripiprazole 2.5 mg/d use. New-onset muscle pain, muscle tenderness, and fatigue were not mentioned. The vitals of the patient, who had been admitted urgently several times due to somatic complaints recently and whose physical examination findings and blood tests were normal, were as follows: fever was evaluated as afebrile (36.5^∘^C), blood pressure 110/60 mm Hg, pulse: 68 beats/min, Oxygen saturation: 99%. No pathology was found in the physical examination of the patient. His weight was 60 kg, and his height was 171 cm (BMI: 20.52 kg/m^2^, height: 25–50 percentile, weight 10–25). On neurological examination, his consciousness, cooperation, and orientation were evaluated as normal. No reduction in muscle strength, muscle tenderness or rigidity was detected. All abdominal USG, POAG, SDAR, and ECG were found to be normal. On laboratory tests, hemoglobin was: 14.0 g/dL, WBC: 7.91 103/μL, AST: 347 IU/L, ALT: 119 IU/L, LDH: 447 IU/L, CK: 18942 IU/L (Table 1). Kidney function tests, ion parameters, sedimentation rate, coagulation parameters, cardiac parameters, and complete urinalysis were within normal limits. He stated that for the last two days, his urine was a little darker than before. There was no history of repetitive injections, infections, heavy sports, or alcohol and substance use that could have caused the elevated CK. Viral infection tests (HAV, HBV, HCV, EBC, HIV, CMV) and urine toxic substance screening tests were negative. No problem was detected in the result of the TANDEM MS (carnitine/acylcarnitine analysis) test. The patient did not have any signs of serotonin syndrome or neuroleptic malignant syndrome. As a result of the findings and examinations of the patient, the condition was diagnosed as rhabdomyolysis related to the use of aripiprazole. The aripiprazole treatment was withheld immediately, and intravenous hydration therapy was initiated while he was being monitored in the emergency service. Control CK values ​​decreased within 5 days as 14689 IU/L, 11669 IU/L, 7954 IU/L, 4697 IU/L, 626 IU/L with daily examinations (Table 1). The symptoms of weakness, nausea, dizziness resolved on admission and urine color returned to normal. He was discharged from the emergency service on the 5th day of monitoring. All laboratory examinations performed on the 10th day for control purposes were normal and the CK value returned to normal values ​​as 122 IU/L (Table 1).

**Table 1:** Biochemical Parameters

**Day of measurement**	**CK (IU/L)**	**AST(IU/L)**	**ALT(IU/L)**	**LDH(IU/L)**	**ALP(IU/L)**
1st	**18942**	**347**	**119**	**447**	73
2st	**14689**	**283**	**104**	**-**	-
3rd	**11669**	**236**	**98**	**-**	62
4th	**7954**	**198**	**94**	**-**	71
5th	**4697**	**131**	**79**	**193**	66
6th	**626**	**44**	**64**	**154**	86
10th	122	17	18	-	-

*Measurements above the reference values are shown in bold CK, creatine kinase; AST, aspartate aminotransferase; ALT, alanine aminotransferase; LDH, lactate dehydrogenase; ALP, alkaline phosphatase.

## Discussion

 Rhabdomyolysis is a serious clinical condition that can lead to acute renal failure and hyperkalemia, as well as electrolyte disturbances, compartment syndrome, disseminated intravascular coagulation, and peripheral neuropathy because of the destruction of muscle cells and the introduction of intracellular materials into the systemic circulation. Rhabdomyolysis may develop due to trauma, special exercise, drug use, substance use and infections. Clinically, symptoms such as fever, nausea-vomiting, and muscle weakness can be seen. They may have different appearances ranging from asymptomatic muscle enzyme elevation to serum electrolyte imbalance and acute kidney injury. The diagnosis is confirmed when the serum CK level is > 1000 U/L or at least five times the upper limit of normal.

 Rhabdomyolysis due to antipsychotic drug use is a life-threatening side effect that requires hospitalization. Rhabdomyolysis can be seen alone or may be associated with the neuroleptic malignant syndrome. Although the exact mechanism of its occurrence due to use of medication is not known, it is thought to be associated with serotonergic or dopaminergic blockade.^7^

 Antipsychotics can increase cell membrane permeability in skeletal muscles due to serotonin antagonism in susceptible patients. Increased CK permeability in the sarcolemma and reduction of glucose uptake is observed due to blockade of this receptor. Another possible mechanism is that due to dopaminergic blockade in the nigrostriatal pathway, involuntary movements such as rigidity, parkinsonism, and akathisia are observed, causing CK elevations. Some cases of rhabdomyolysis reported with typical antipsychotics such as haloperidol, fluphenazine, thioridazine, and loxapine developed due to drug overdose. Rhabdomyolysis cases caused by clozapine, quetiapine, risperidone, and olanzapine are also available in the literature.^8^ Cases in children and adolescents are usually related to olanzapine use.^9^ In a study, 106 rhabdomyolysis cases were examined, and it was reported that they were caused by quetiapine use in 8 cases. Alcohol and substance use were reported in some cases.^10^

 Aripiprazole is a second-generation atypical antipsychotic acting as a partial agonist of dopamine D2 and D3 receptors and serotonin 5-HT1A receptors and a partial antagonist of serotonin 5-HT2A receptors.^11^ The mechanism of rhabdomyolysis that develops on account of aripiprazole is explained by the antagonist effect on the receptor; however, it has not been fully elucidated. It has an antagonistic effect on the 5-HT2A receptors found in the skeletal muscle.^3^ According to this hypothesis, in susceptible patients, aripiprazole has been suggested to antagonize serotonin 5-HT2A, rapidly increasing glucose uptake into myocytes, causing changes in the sarcolemma structure and increasing CPK permeability, leading to an increase in the permeability of myocyte cell membrane.^12,13^ Inhibition of 5-HT2A receptors by aripiprazole can increase traumatic muscle damage by compromising myocyte energy metabolism and calcium homeostasis.^14^

 A few cases of rhabdomyolysis triggered by aripiprazole use were found in the literature. The first case was a 31-year-old male patient with a diagnosis of schizophrenia reported from Taiwan. Quetiapine was discontinued and aripiprazole treatment was initiated owing to increased sleepiness in the case who was using quetiapine. He was diagnosed with drug-induced rhabdomyolysis on the 30th day of aripiprazole use of 15 mg/d.^12^ The other case was an 87-year-old female patient with hypertension and renal failure, with a diagnosis of bipolar disorder reported from Italy. Aripiprazole was added to the patient’s treatment regimen, who was using clozapine and valproate treatment a few months ago. After a traumatic hip fracture, she was evaluated as having rhabdomyolysis resulting from the use of aripiprazole, as CK was found to be high in the hospital admission examinations.^13^ A 37-year-old male patient diagnosed with schizophrenia, bipolar disorder and mild developmental delay was administered 400 IU aripiprazole intramuscularly. While he was still taking 10 mg aripiprazole and 10 mg olanzapine orally, he was diagnosed with rhabdomyolysis on the 22nd day of injection^15^ A 30-year-old female patient with a diagnosis of compartment syndrome, factitious disorder, self-mutilation and borderline personality disorder was followed up with aripiprazole 20 mg/day for 4 years and was diagnosed with rhabdomyolysis as a result of physical activity.^16^ In a retrospective study of rhabdomyolysis in children, a case of rhabdomyolysis due to aripiprazole use was reported.^6^

 This is a rare case of rhabdomyolysis in children and adolescents due to the adverse effects of aripiprazole. The short duration of use and low dose exposure are the features that make this case prominent. It should be kept in mind that even a drug considered to be safe compared to other antipsychotics in terms of such side effects may have individual predispositions and rhabdomyolysis may develop at a young age at low doses. Therefore, observing basal blood levels before starting antipsychotic treatment in the child-adolescent age group and during the follow-up process, nonspecific symptoms such as muscle fatigue, pain, and weakness, which are likely to be missed because they are similar to the symptoms of the disease, should be considered, keeping in mind that rhabdomyolysis may develop, and blood tests and physical complaints should be followed up. Patients may experience symptoms such as fever, nausea, vomiting, confusion, agitation, delirium, and anuria.^17^ During antipsychotic therapy, it is crucial to monitor for unexpected symptoms in each patient. Abdominal pain is an uncommon finding of rhabdomyolysis, but 12% of 191 children and adolescents with rhabdomyolysis for any reason presented with abdominal tenderness. Symptoms such as abdominal pain, muscle pain, weakness and dark urine with the use of antipsychotics should be followed up. These symptoms may be precursors of rhabdomyolysis, which may cause acute renal failure. If there is a dose increase, drug regimen change, or use of multiple antipsychotics, newly developed symptoms should be screened. Also intramuscular injections, alcohol-substance use, exposure to heavy sports activities, and the presence of seizures are risky for rhabdomyolysis.^8^

 Finally, the mechanism of rhabdomyolysis caused by antipsychotics has not been fully elucidated. Since non-specific symptoms of rhabdomyolysis can be observed in children and adolescents, symptoms should be monitored once treatment begins, especially for 2 months and in presence of risk factors.
